# Supplementary material for: Tradeoffs between resources and risks shape the responses of a large carnivore to human disturbance
Source: Commun Biol. 2023 Oct 17;6:986. doi: 10.1038/s42003-023-05321-z (PMC10582050; doi:10.1038/s42003-023-05321-z)
Supplement: Supplementary file 2 — Supplementary Materials [file 42003_2023_5321_MOESM2_ESM.pdf]

Supplementary Information

Tradeoffs between resources and risks shape the responses of a large carnivore to human disturbance

Kirby L . Mills[1]*, Jerrold L. Belant[2], Maya Beukes[3], Egil Dröge[4,5], Kristoffer T. Everatt[6,7,8], Robert Fyumagwa[9], David S. Green[10], Matt W. Hayward[11,12,13], Kay E. Holekamp[14,15], F.G.T. Radloff[16], Göran Spong[17], Justin P. Suraci[18], Leanne K. Van der Weyde[19,20], Christopher C. Wilmers[21], Neil H. Carter[22]**, Nathan J. Sanders[1]**

[1]Department of Ecology and Evolutionary Biology, University of Michigan, Ann Arbor, MI, USA
[2]Department of Fisheries and Wildlife, Michigan State University, East Lansing, MI, USA
[3]Senckenberg Research Institute and Nature Museum, Terrestrial Zoology, Frankfurt, Germany
[4]WildCRU, Department of Biology, University of Oxford, Tubney, UK
[5]Zambian Carnivore Programme, Mfuwe, Zambia
[6]Panthera, New York, USA
[7]Centre for African Conservation Ecology, Nelson Mandela University, Port Elizabeth, South Africa
[8]Greater Limpopo Carnivore Programme, Limpopo, Mozambique
[9]Wildlife Conservation Initiative, Arusha, United Republic of Tanzania
[10]Institute for Natural Resources, Portland State University, Portland, OR, USA
[11]Conservation Science Research Group, School of Environmental and Life Science, University of Newcastle, Callaghan, Australia
[12]Centre for African Conservation Ecology, Nelson Mandela University, Qgeberha, South Africa
[13]Centre for Wildlife Management, University of Pretoria, Tshwane, South Africa
[14]Department of Integrative Biology, Michigan State University, East Lansing, MI, USA
[15]Program in Ecology, Evolutionary Biology, and Behavior, Michigan State University, East Lansing, Michigan
[16]Department of Conservation and Marine Sciences, Faculty of Applied Sciences, Cape Peninsula University of Technology, Cape Town, South Africa
[17]Molecular Ecology Group, SLU, 901 83 UMEÅ, Sweden
[18]Conservation Science Partners, Inc., Truckee, California, USA
[19]Cheetah Conservation Botswana, Gaborone, Botswana
[20]San Diego Zoo Institute for Conservation Research, Escondido, CA, USA
[21]Environmental Studies Department, University of California, Santa Cruz, California, USA
[22]School for Environment and Sustainability, University of Michigan, Ann Arbor, MI, USA

*Corresponding author: Kirby L. Mills, kimills@umich.edu
**These authors jointly supervised this work.

Keywords: *Panthera leo*, risk-averse foraging, landscape of fear, human-wildlife coexistence, meta-regression, NDVI, pastoralism, case study

Supplemental Methods & Results

Analysis for publication bias: We assessed the presence of publication bias in the spatial and temporal lion response datasets with Egger regression tests of asymmetry for funnel plots that map effect size residuals against the corresponding sampling variances (Figure S5). While spatial lion responses showed symmetrical residuals (z = 0.452, p = 0.652), the regression test of temporal lion responses did suggest publication bias with significant asymmetry (z = -4.12, $p < 0.001$). However, tests for Rosenberg's fail-safe number revealed that the number of studies with null responses necessary to change the significance of our results was many times higher than the actual sample sizes of the models. To increase the observed significance of the mixed-effects models to > 0.05 would require including an additional 249 null spatial effect sizes and 191 null temporal effect sizes. The very large number of studies needed to nullify the observed significance of the mixed-effects models thus suggests that publication bias is unlikely to have substantially impacted our results. In addition, many studies included in this analysis were not explicitly designed to detect lion responses to human disturbance, which lessens the risk of publication biases that could compromise our results.

Spatial autocorrelation of effect sizes: We tested the observed effect sizes of human disturbance on lion activity for spatial autocorrelation using Moran's *I* but found no significant autocorrelation for either spatial (p = 0.20) or temporal effect sizes (p = 0.99).

Figure S1 – Flow chart outlining the procedure for identifying and including relevant studies for use in the meta-analysis. Numbers indicate the number of publications produced by the preceding step that were used in the subsequent step (e.g., Web of Science search resulted in 344 publications that were then screened based on the publication title).

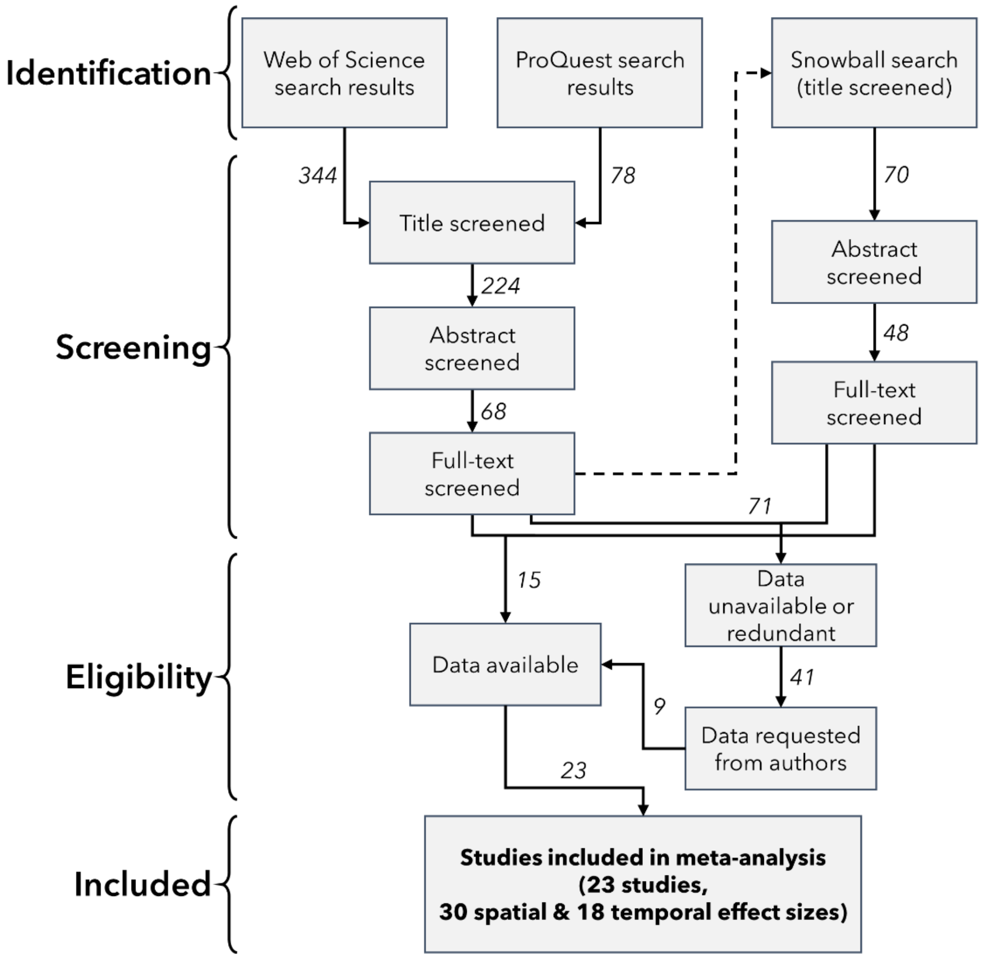

Figure S2 – Decision tree chart illustrating the process for extracting lion activity and human disturbance data from included studies.

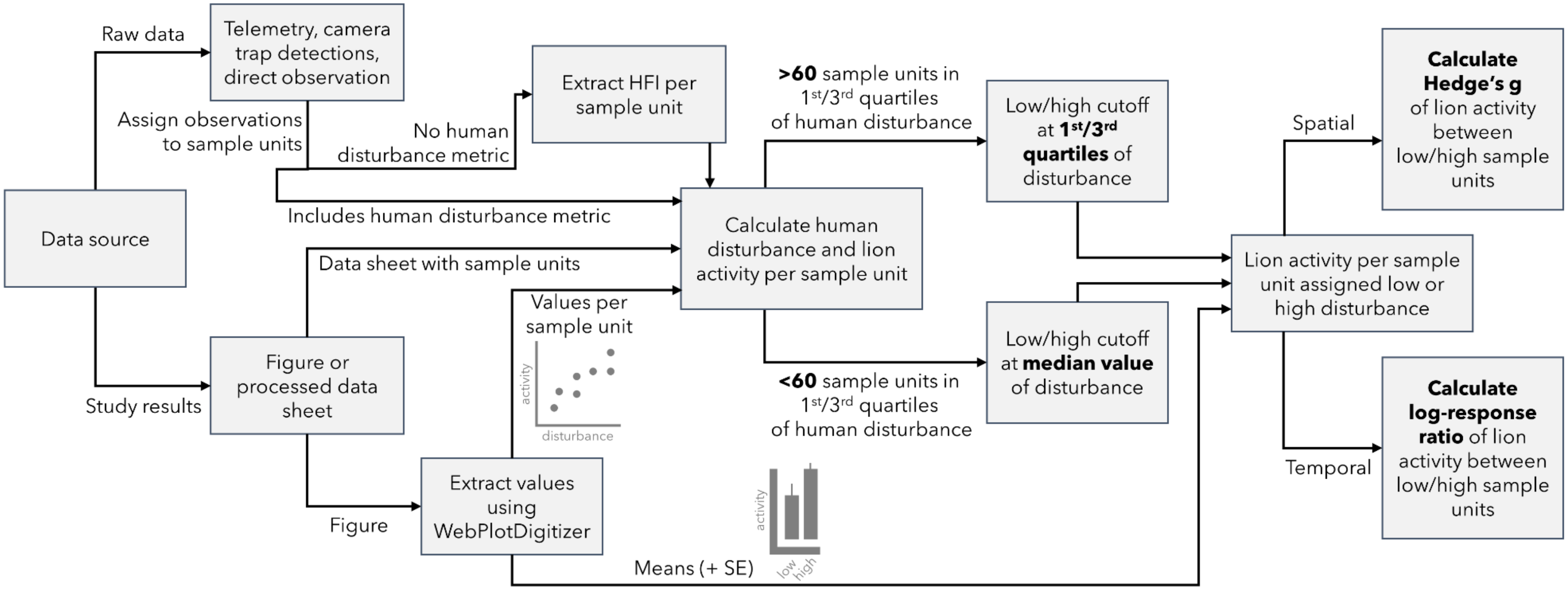

Figure S3 – Relationship between monthly average precipitation and Normalized Difference Vegetation Index (NDVI) values at African study sites between 2000-2019. Each line represents the linear model for a study site, each point a monthly value of precipitation and NDVI. A linear model including study site as an interaction term with NDVI indicated significant positive relationships between monthly NDVI and precipitation at all but one study site (see Table S3 for correlation coefficients by study site).

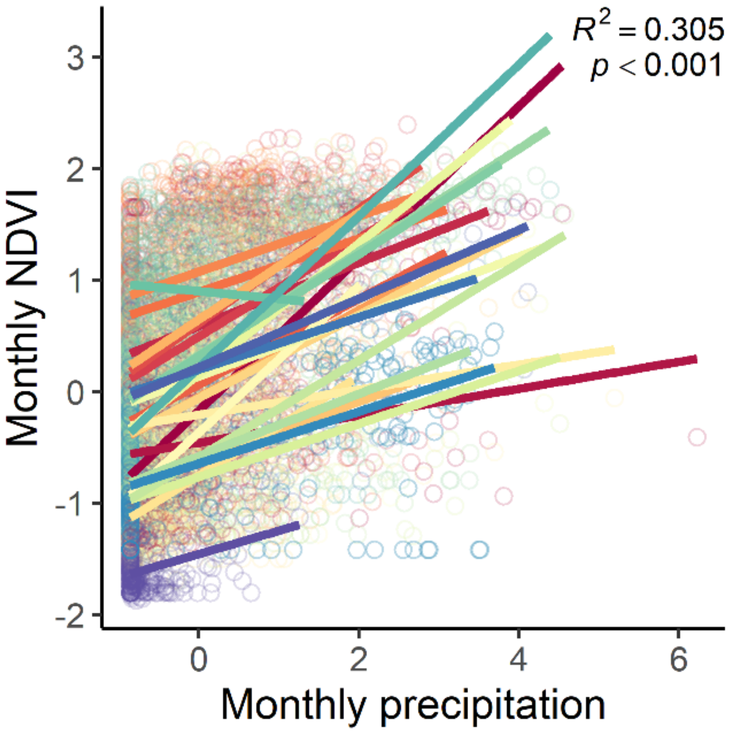

Figure S4 – Distribution of effect sizes of lion responses to human disturbance by categorical study characteristics: the type of lion observation data based on methods used to monitor lions, whether the study area was fenced or un-fenced, the type of human disturbance that was measured in the study and/or used to calculate effect sizes, and the period of the year in which lions were monitored. Negative values on both y-axes indicate lion avoidance of human disturbance in time and space. All variables are scaled and centered. *P*-values show the result of ANOVA tests of the variables on spatial (green) and temporal (purple) effect sizes, and significant effects ($p < 0.05$) are denoted by a star. The human footprint was considered to represent 'both' types of human disturbance, because the metric is a combination of many human disturbances, including human population size, land use types, and built infrastructure. SMD = standardized mean difference; RR = log response ratio.

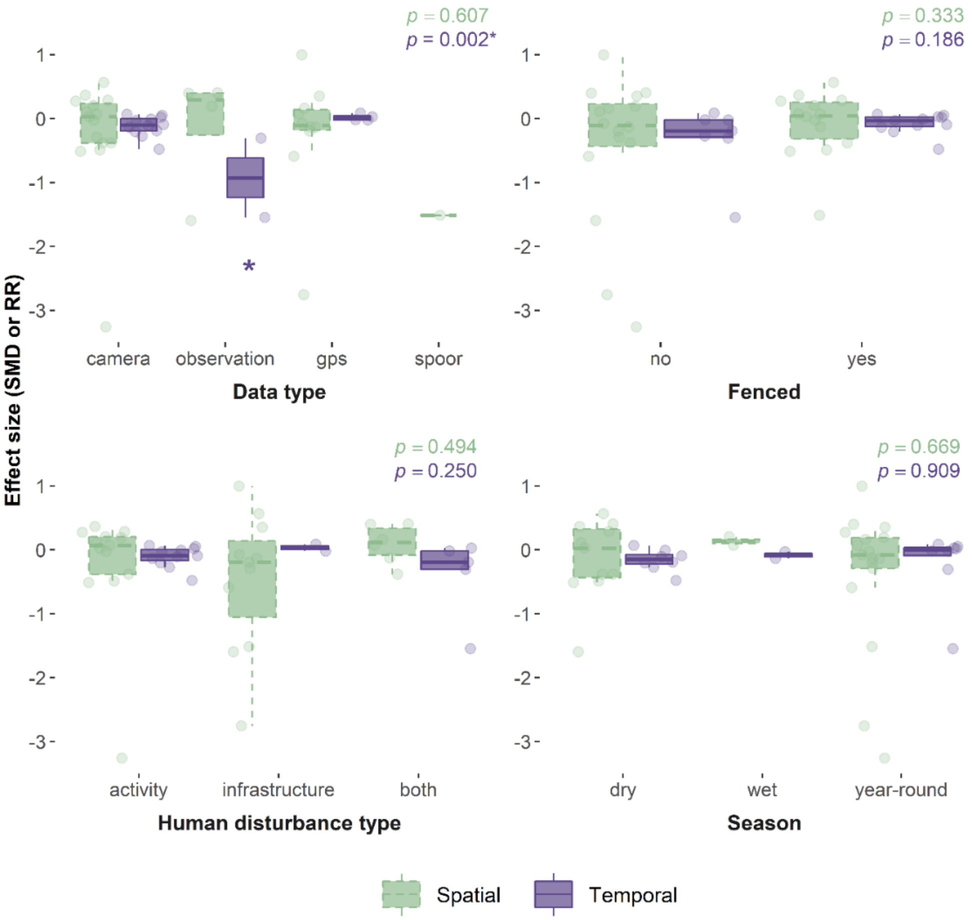

Figure S5 - Distribution of effect sizes of lion responses to human disturbance by continuous study characteristics: the median date of the study period, the log-transformed size of the study area measured in sq-km, and the log-transformed duration of the study period in months. Negative values on both y-axes indicate lion avoidance of human disturbance in time and space. All variables are scaled and centered. *P*-values show the result of linear models for each variable's relationship with spatial (green) and temporal (purple) effect sizes, and significant effects ($p < 0.05$) are denoted by a star. SMD = standardized mean difference; RR = log response ratio.

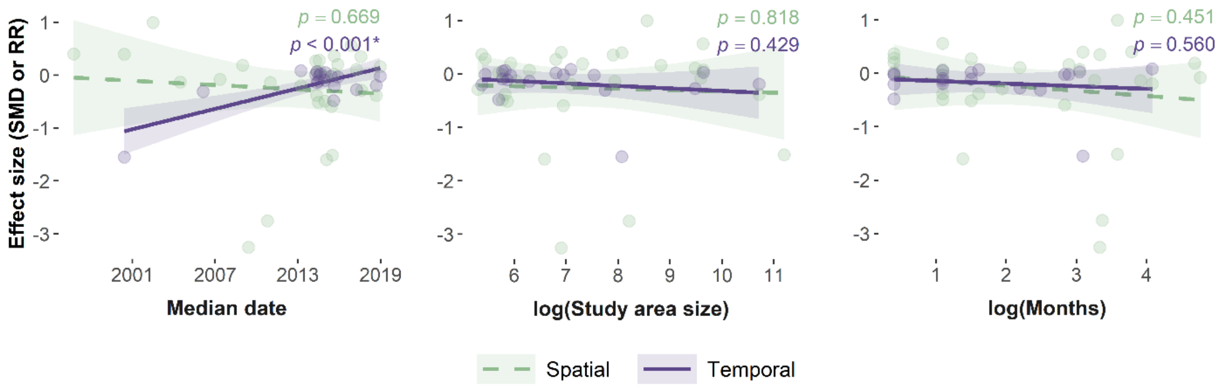

Figure S6 – Funnel plots for the lowest AIC_c mixed-effects models of lion spatial and temporal responses to human disturbance, comparing the effect size residuals to their corresponding sampling variance. Egger regression tests were used to test funnel plot asymmetry to check for possible publication bias.

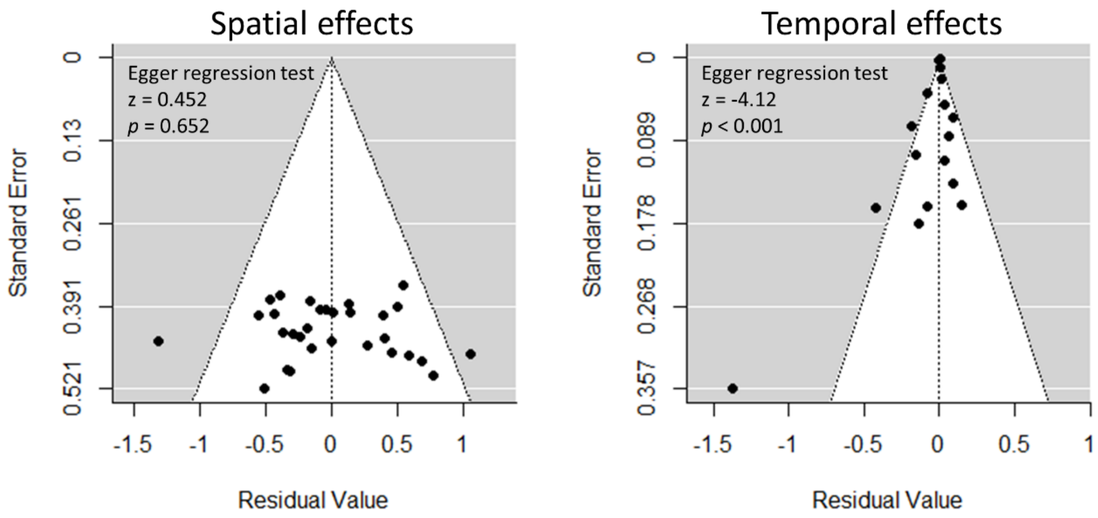

Table S1 – Descriptions of the locations and study periods for each study site included in our meta-analysis.

Study reference	Study area	Country	Study area size (km^2)	Latitude, Longitude	Season	Study period	Duration (months)
Beukes et al. 2017 (1)	Kgalagadi Transfrontier Park	South Africa	15,396	-25.38, 20.38	year-round	2013-2015	21
Chaudhary et al. 2020 (2)	Gir Protected Area	India	200	21.14, 70.83	year-round	2017-2018	7
Dolrenry 2013 (3)	Amboseli-Tsavo Ecosystem	Kenya	3,684	-2.63, 37.26	year-round	2009-2011	29
Dröge et al. 2020 (4)	Kafue National Park	Zambia	2,702	-14.43, 25.93	year-round	2016-2018	28
Dröge et al. 2017 (5)	Liuwa Plain National Park	Zambia	1,200	-14.5, 22.5	year-round	2010-2015	59
Everatt et al. 2019 (7)	Greater Limpopo Transfrontier Conservation Area	Mozambique	73,000	-22.93, 32.31	year-round	2014-2016	36
Gogoi et al. 2020 (8)	Gir Protected Area	India	725	21.14, 70.83	dry	2014-2015	4
Green et al. 2018 (9)	Masai Mara National Reserve	Tanzania	1,500	-1.47, 35.07	year-round	2004-2013	108
Hayward & Hayward 2006 (10)	Addo Elephant National Park	South Africa	420	-33.47, 25.75	year-round	2003-2005	26
Loveridge et al. 2017 (11)	Hwange National Park	Zimbabwe	14,650	-19, 26.5	year-round	2002-2012	117
Maddox 2003 (12)	Serengeti-Mara ecosystem	Tanzania	3,200	-2.61, 35.22	year-round	1999-2001	22
Miller et al. 2018 (13)	Hluhluwe-iMfolozi Park	South Africa	900	-28.22, 31.95	year-round	2013-2015	4.5
	KwaZulu Private Game Reserve	South Africa	377	-27.57, 31.66	wet	2015	1.5
	Makalali Private Game Reserve	South Africa	323	-24.21, 30.63	year-round	2014-2015	3
	Phinda Private Game Reserve	South Africa	230	-27.81, 32.35	dry	2014	1.5
	Tembe Elephant Park	South Africa	299	-26.95, 32.44	dry	2015	1.5
	Timbavati Private Game Reserve	South Africa	541	-24.4, 31.31	wet	2013-2014	3
	uMkhuze Game Reserve	South Africa	353	-27.65, 32.15	dry	2013-2015	4.5

Miller et al. 2018 (13)	Venetia-Limpopo Nature Reserve	South Africa	321	-22.35, 29.31	dry	2014-2015	3
	Welgevonden Private Game Reserve	South Africa	339	-24.31, 27.83	dry	2013-2015	5
	Zululand Rhino Reserve	South Africa	216	-27.77, 32.11	dry	2015	1.5
Mills et al. 2020 (14);<br>Mills & Harris 2020 (15)	W-Arly-Pendjari	Burkina Faso, Niger	13,100	11.5, 1.5	dry	2016-2018	9
Mogensen et al. 2011 (16)	Masai Mara National Reserve and Koyaki Ranch	Kenya	2,323	11.5, 1.5	year-round	2005-2006	12
Oriol-Cotterill et al. 2015 (17)	Laikipia County	Kenya	2,800	-1.44, 35.21	year-round	2009-2012	50
Schooler et al. 2022 (18)	Serengeti ecosystem	Tanzania	6,800	-2.67, 34.58	year-round	2018-2019	18
Schuette et al. 2013 (19)	Southern Rift Valley	Kenya	1,000	0.57, 36.67	year-round	2008-2010	28
Searle et al. 2021 (20)	Ruaha-Rungwa Landscape	Tanzania	45,000	-1.92, 36.13	dry	2018-2019	6
Spong 2002 (21)	Selous Game Reserve	Tanzania	1,000	-7.4, 34.8	dry	1994-1999	44
Suraci et al. 2019 (22)	Laikipia District	Kenya	1,040	-7.58, 38.25	year-round	2014-2016	17
Valeix et al. 2012 (23)	Makgadikgadi Pans National Park	Botswana	5,200	0.44, 36.81	year-round	2001-2003	36
Van der Weyde et al. 2018 (24)	Okwa Wildlife Management Area	Botswana	15,290	-20.5, 23	dry	2016	3

Table S2 – Descriptions of lion and human data and method of data extraction for each study included in our meta-analysis. In data extraction descriptions: S = spatial data extraction, T = temporal data extraction, HFI = human footprint index.

Study	Data type	Lion activity metric	Human disturbance metric	Data extraction description	ES type	ES	Var.	Low disturbance Mean (SD)	Low disturbance N	High disturbance Mean (SD)	High disturbance N
Beukes et al. 2017 (1)	GPS	detections	human footprint	Created grid across lion locations using average daily displacement for grid size (~4.9-km), designated grid cells as low/high human disturbance based on average HFI value per grid cell (1st/3rd quartiles). *S:* Calculated average number of observations per grid cell. *T:* Calculated the proportion of fixes representing active lion movement during day/night.	SMD	0.07	0.021	191.76 (290.06)	96	222.38 (520.37)	96
					RR	0.03	0.000	0.76	3402	0.74	3929
Chaudhary et al. 2020 (2)	camera	detections	distance to residential	Sample units assigned low/high human disturbance using average distance to settlement. Calculated average number of lion observations per camera station.	SMD	-0.29	0.081	5.16 (6.18)	31	3.24 (7.06)	21
Dolrenry 2013 (3)	GPS, observation	occupancy	building density	Lion occupancy was extracted from 44 equidistant points along line in Figure 3.3, using WebPlotDigitizer. Lion density was assigned to high/low categories based on median boma density.	SMD	-2.75	0.170	0.88 (0.05)	22	0.61 (0.12)	24
Dröge et al. 2020 (4)	GPS	detections	human footprint	Converted lion tracks from supplemental data to points at track vertices. Created 1000m grid across lion locations, calculated average	SMD	0.35	0.008	3.59 (3.73)	244	6.16 (9.63)	244

				number of observations per grid cell, designated lion locations as low/high human disturbance based on average HFI value per grid cell (1st/3rd quartiles).							
Dröge et al. 2017 (5)	GPS	GPS locations	distance to residential	Created 1000m grid across lion locations, used 1st and 3rd quartiles of distance to village for low/high disturbance categories. *S:* Calculated average number of fixes and their distances to village within each grid cell *T:* Calculated the proportion of fixes representing active lion movement during day/night.	SMD	-0.20	0.010	11.27 (37.66)	202	5.73 (13.06)	201
					RR	0.09	0.001	0.83	1167	0.77	473
Everatt et al. 2019 (7)	spoor	occupancy	distance to residential	Averaged lion occupancy across sample units; sample units assigned high/low human disturbance using median of distance to village variable.	SMD	-1.51	0.050	0.67 (0.21)	52	0.28 (0.3)	51
Gogoi et al. 2020 (8)	observation	spatial density	distance to residential	Lion density and distance to human habitation values extracted from Fig S5. Sample units assigned high/low human disturbance using 1st and 4th quartiles of distance to habitation variable. Calculated average lion density for high/low categories.	SMD	-1.60	0.066	0.41 (0.84)	40	-0.87 (0.74)	40

Green et al. 2018 (9)	observation	detections	land use type	Designated Talek West as high disturbance, other areas as low disturbance based on site descriptions in text. Created 1000m grid across lion locations, calculated average number of observations per 100 sample days per grid (4 years for low disturbance, 7 years for high disturbance based on text descriptions).	SMD	0.19	0.018	0.44 (0.67)	137	0.6 (1.04)	94
Hayward & Hayward 2006 (10)	GPS	detections	human footprint	Created grid across lion locations based on avg daily displacement (~5.5-km), designated grid cells as low/high human disturbance based on average HFI value per grid cell (1st/3rd quartiles). Calculated average number of lion observations per grid cell.	SMD	-0.13	0.020	17.18 (18.58)	96	14.68 (19.24)	110
Loveridge et al. 2017 (11)	GPS	space use	distance to residential	Designated low/high disturbance using median distance to village (22714m). Calculated the average the number of observations among individuals in low vs high human disturbance locations.	SMD	-0.08	0.032	16.69 (12.49)	62	15.71 (12.52)	62
Maddox 2003 (12)	observation	density, behavioral observations	land use type	Characterized Loliondo as high human disturbance and NCA and Serengeti as low human disturbance based on site descriptions in text. *S:* Extracted	SMD	0.40	0.021	0.28 (0.1)	70	0.32 (0.13)	152

				average lion densities (SE) from Figure 26, calculated SD using sample sizes in text. *T:* Extracted sample sizes and proportion active from Figure 41, converted proportions to counts to calculate proportion nocturnal activity.	RR	-1.55	0.129	0.17	47	0.80	10
Miller et al. 2018 (13)	camera	detections	human trap success	Calculated trap success (detections/100 trap-nights) for humans & lions for each camera stations from supplemental raw data. Camera stations assigned low/high human disturbance using median values of human trap-success. *S:* Lion trap success averaged for disturbance categories. *T:* Calculated the proportion of lion detections during day/night.	SMD	0.27[1]	0.054	7.17 (7.1)	32	9.9 (11.45)	45
						0.20[2]	0.097	4.2 (5.13)	29	5.98 (12.79)	16
						-0.03[3]	0.052	22.5 (25.55)	38	21.77 (29.07)	39
						0.28[4]	0.097	4.84 (8.09)	23	7.42 (9.76)	19
						0.03[5]	0.126	11.67 (8.45)	15	11.9 (9.22)	17
						0.07[6]	0.061	3.16 (6.11)	31	3.53 (4.51)	35
						-0.51[7]	0.042	2.92 (5.57)	53	0.71 (1.74)	45
						-0.49[8]	0.063	5.38 (4.9)	33	3.23 (3.7)	32
						-0.38[9]	0.041	7.53 (7.09)	54	4.76 (7.32)	45
						0.37[10]	0.136	4.39 (6.22)	15	13.48 (33.65)	15

					RR	0.02[1]	0.003	0.85	101	0.84	196
						-0.03[2]	0.020	0.66	56	0.68	44
						0.05[3]	0.004	0.63	288	0.60	286
						-0.01[4]	0.013	0.73	49	0.74	62
						-0.48[5]	0.027	0.39	77	0.63	89
						-0.14[6]	0.011	0.72	50	0.83	63
						-0.11[7]	0.002	0.90	68	1.00	14
						-0.10[8]	0.006	0.81	79	0.89	46
						0.07[9]	0.007	0.69	207	0.64	109
						-0.20[10]	0.029	0.59	29	0.72	89
Mills et al. 2020 (14)	camera	occupancy	occupancy	Camera grids were designated as being in low or high human disturbance sites according to human disturbance designations in Mills & Harris 2020 (15). Calculated average lion occupancy between low/high disturbance grids.	SMD	0.11	0.020	0.56 (0.24)	94	0.58 (0.25)	110
Mills & Harris 2020 (15)	camera	detections	occupancy	Calculated the proportion of lion detections during day/night between low and high human camera stations designated in study. Nocturnal times determined as described in the study text. Uses same raw data set as Mills et al. 2020 (14).	RR	-0.27	0.032	0.53	36	0.69	62
Mogensen et al. 2011 (16)	observation	activity type	land use type	Designated pride A and B as low disturbance, pride C as high disturbance based on study site descriptions in text. Calculated	RR	-0.31	0.038	0.66	16	0.90	18

				number of active and nocturnal detections from Figure 2.							
Oriol-Cotterill et al. 2015 (17)	GPS	detections	distance to boma	Extracted and pooled means and SE of distance to boma at 1200 (high human disturbance) and between 2300-0500 (low human disturbance) in Fig. 3. Sample sizes calculated from total # fixes reported, divided by 2 into seasons, then divided into equal numbers of fixes per hour reported. Values inverted to represent lion use and avoidance in accordance with ES interpretations.	SMD	-0.14	0.001	-2.86 (2.7)	1340 4	-3.2 (0.29)	2234
Schooler et al. 2022 (18)	GPS	detections	human footprint	Created grid across lion locations using average daily displacement for grid size (~2.3-km), designated grid cells as low/high human disturbance based on average HFI value per grid cell (1st/3rd quartiles). *S:* Calculated average number of observations per grid cell. *T:* Calculated the proportion of fixes representing active lion movement during day/night.	SMD	0.16	0.012	109.91 (232.8)	166	154.11 (304.66)	166
					RR	-0.02	0.000	0.63	2953	0.65	3531
Schuette et al. 2013 (19)	camera	occupancy	land use type	Designated 'CCS' site as low disturbance and 'Grazing Area' site as high disturbance based on site descriptions in text. Extracted the lion occupancy mean and SD for both sites in Figure 4b.	SMD	-3.26	0.097	0.67 (0.27)	40	0.08 (0.06)	57

Searle et al. 2021 (20)	camera	detections	land use type	Classified MBOMIPA WMA as high human disturbance and other sites as low disturbance based on text descriptions. 'Ruaha NP miombo woodland' survey was excluded from analysis because of data issue discussed in text. *S:* Calculated average lion trap success per camera station in low vs. high sample units. *T:* Calculated proportion of lion detections in day/night.	SMD	-0.38	0.082	21.26 (18.2)	67	14.56 (12.58)	15
					RR	-0.19	0.000	0.82	598	1.00	83
Spong 2002 (21)	observation	detections	human footprint	Created 1000m grid across lion locations, and designated grid cells as low/high human disturbance based on the median (among grid cells) of average within-grid cell HFI value. Calculated average number of lion observations per grid cell.	SMD	0.40	0.025	2.29 (2.03)	108	3.64 (4.77)	67
Suraci et al. 2019 (22)	GPS	detections	distance to boma	Created grid across lion locations using average daily displacement for grid size (~3-km), designated grid cells as low/high human disturbance based on average distance to nearest boma per grid cell (1st/3rd quartiles). *S:* Calculated average number of observations per grid cell *T:* Calculated the proportion of fixes representing active lion movement during day/night, as designated by the study text definition of nighttime from 08:00 and 18:00.	SMD	-0.59	0.037	1666.21 (1602.08)	57	719.23 (1571.85)	57
					RR	-0.02	0.000	0.93	2308	0.95	1032

Valeix et al. 2012 (23)	GPS	Jacob's index	distance to boma	Designated 0-6km from cattle-posts as high and >6km as low disturbance based on text descriptions. Extracted mean and 95%CI of Jacob's index of selection, calculated SD from sample sizes described in text.	SMD	1.00	0.069	-0.42 (0.37)	18	-0.02 (0.39)	108
Van der Weyde et al. 2018 (24)	camera	detections	distance to residential	Designated camera stations as low/high human disturbance based on median distance from village. Calculated lion trap success (detections/100 trap-nights) for camera stations from raw data.	SMD	0.56	0.043	0.18 (0.69)	48	1.8 (4.0)	48

Sites in Miller et al. 2018: [1]Hluhluwe-iMfolozi Park, [2]KwaZulu Private Game Reserve, [3]Makalali Private Game Reserve, [4]Phinda Private Game, Reserve, [5]Tembe Elephant Park, [6]Timbavati Private Game Reserve, [7]uMkhuze Game Reserve, [8]Venetia-Limpopo Nature Reserve, [9]Welgevonden Private Game Reserve, [10]Zululand Rhino Reserve

Table S3 - Top performing mixed-effects models and global models used to assess the effects of ecological and anthropogenic local conditions on the magnitude of lion responses (SMD and RR), with model evaluation parameters and estimated coefficients of model variables. to human disturbance. All models within 2 ΔAICc from the lowest AIC_c model were included in the final model set (also used for weighted model averaging [Table S2]). SMD_w = average weighted standardized mean difference (REM intercept); RR_w = average weighted log response ratio (REM intercept); HFI_{sp} = spatial variation in human footprint index; CAT_a = average cattle production; $NDVI_{sp}$ = average spatial variation in NDVI; $NDVI_{tm}$ = temporal variation in NDVI; $NDVI_a$ = average overall NDVI. *coefficient 95% CI significantly different from 0.

							Model Coefficients (95% CI)						
**Mixed-effects models**	**AIC_c**	**ΔAIC_c**	**Model weight**	**R^2**	**$\hat{\tau}^2$ 95% CI**	**I^2 95% CI**	**Average ES**	**HFI_a**	**HFI_{sp}**	**CAT_a**	**$NDVI_a$**	**$NDVI_{tm}$**	**$NDVI_{sp}$**
**Spatial responses**							**SMD_w**						
$HFI_{sp} + NDVI_{sp} + NDVI_{tm}$	52.95	0	0.45	0.76	0.108-0.449	82.2-95.0%	-0.268* (-0.433, -0.103)	-	0.286* (0.004, 0.092)	-	-	0.218* (0.034, 0.402)	-0.893* (-1.114, -0.671)
$HFI_{sp} + CAT_a + NDVI_{sp} + NDVI_{tm}$	53.34	0.38	0.37	0.79	0.099-0.432	80.6-94.8%	-0.279* (-0.437, -0.121)	-	0.243* (0.052, 0.434)	-0.158 (-0.339, 0.024)	-	0.235* (0.059, 0.411)	-0.820* (-1.048, -0.592)
$HFI_{sp} + NDVI_{sp}$	54.79	1.83	0.18	0.69	0.132-0.491	85.0-95.5%	-0.254* (-0.437, -0.071)	-	0.245* (0.034, 0.457)	-	-	-	-0.819* (-1.050, -0.589)
Global Model	59.58	6.63		0.76	0.105-0.474	80.3-94.9%	-0.267* (-0.431, -0.103)	-0.081 (-0.262, 0.100)	0.233* (0.041, 0.426)	-0.162 (-0.347, 0.022)	-0.021 (-0.192, 0.151)	0.236* (0.056, 0.416)	-0.783* (-1.049, -0.517)
**Temporal responses**							**RR_w**						
$HFI_{sp} + CAT_a + NDVI_a + NDVI_{sp}$	-6.29	0	0.32	1	0.007-0.251	66.7-98.7%	-0.068* (-0.104, -0.033)	-	0.061* (0.039, 0.084)	-0.102* (-0.160, -0.044)	-0.015* (-0.027, -0.002)	-	-0.061* (-0.076, -0.045)
$HFI_{sp} + CAT_a + NDVI_{sp}$	-6.12	0.17	0.29	0.95	0.005-0.219	73.1-99.1&	-0.075* (-0.115, -0.036)	-	0.056* (0.028, 0.084)	-0.118* (-0.175, -0.061)	-	-	-0.053* (-0.073, -0.032)
CAT_a	-5.42	0.87	0.2	0.38	0.006-0.183	81.7-99.3%	-0.089* (-0.142, -0.036)	-	-	-0.095* (-0.160, -0.029)	-	-	-
$CAT_a + NDVI_{sp}$	-5.27	1.02	0.19	0.61	0.005-0.189	76.4-99.2%	-0.07* (-0.119, -0.021)	-	-	-0.074* (-0.134, -0.015)	-	-	-0.037* (-0.075, -0.001)
Global Model	5.84	12.13		0.62	0.006-0.293	61.2-98.7%	-0.026 (-0.066, 0.013)	0.013 (-0.016, 0.042)	0.046* (0.016, 0.077)	-0.089* (-0.151, -0.028)	-0.022* (-0.039, -0.005)	0.034 (-0.023, 0.092)	-0.058* (-0.081, -0.035)

Table S4 – Results of tests of correlation between monthly precipitation vs. NDVI at African study sites. Study sites in Gir National Forest, India, were excluded due to the spatial extent of the monthly precipitation data. *Person's *r* coefficient *p*-value < 0.05.

Study site (Study ID)	Study period	Pearson's *r*	*p*-value
Addo Elephant National Park (10)	2003-2005	-0.055	0.387
Amboseli-Tsavo Ecosystem (3)	2009-2011	0.379*	<0.001
Greater Limpopo Transfrontier Conservation Area (7)	2014-2016	0.262*	<0.001
Hluhluwe-iMfolozi Park (13)	2013-2015	0.350*	<0.001
Hwange National Park (11)	2002-2012	0.631*	<0.001
Kafue National Park (4)	2016-2018	0.683*	<0.001
Kgalagadi Transfrontier Park (1)	2013-2015	0.326*	<0.001
KwaZulu Private Game Reserve (13)	2015	0.498*	<0.001
Laikipia County (17)	2009-2012	0.154*	0.014
Laikipia District (22)	2014-2016	0.192*	0.002
Liuwa Plain National Park	2010-2015	0.576*	<0.001
Makalali Private Game Reserve (13)	2014-2015	0.346*	<0.001
Makgadikgadi Pans National Park (23)	2001-2003	0.567*	<0.001
Masai Mara National Reserve and Koyaki Ranch (16)	2005-2006	0.336*	<0.001
Masai Mara National Reserve (9)	2004-2013	0.354*	<0.001
Okwa Wildlife Management Area (24)	2016	0.484*	<0.001
Phinda Private Game Reserve (13)	2014	0.278*	<0.001
Ruaha-Rungwa Landscape (20)	2018	0.733*	<0.001
Selous Game Reserve (21)	1994-1999	0.579*	<0.001
Serengeti ecosystem (18)	2018-2019	0.582*	<0.001
Serengeti-Mara ecosystem (12)	1999-2001	0.499*	<0.001
Southern Rift Valley (19)	2008-2010	0.418*	<0.001
Tembe Elephant Park (13)	2015	0.329*	<0.001
Timbavati Private Game Reserve (13)	2013-2014	0.353*	<0.001
uMkhuze Game Reserve (13)	2013-2015	0.428*	<0.001
Venetia-Limpopo Nature Reserve (13)	2014-2015	0.319*	<0.001
W-Arly-Pendjari (14, 15)	2016-2018	0.794*	<0.001
Welgevonden Private Game Reserve (13)	2013-2015	0.498*	<0.001
Zululand Rhino Reserve (13)	2015	0.406*	<0.001

Supplementary References

1. M. Beukes, F. G. T. Radloff, S. M. Ferreira, Estimating lion's prey species profile in an arid environment. *J. Zool.* **303**, 136–144 (2017).
2. R. Chaudhary, N. Zehra, A. Musavi, J. A. Khan, Evaluating the effect of ecological and anthropogenic variables on site use by sympatric large carnivores in Gir protected area, Gujarat, India. *Wildlife Biol.* **2020** (2020), doi:10.2981/wlb.00696.
3. S. Dolrenry, thesis (2013).
4. E. Dröge, S. Creel, M. S. Becker, A. J. Loveridge, L. L. Sousa, D. W. Macdonald, Assessing the performance of index calibration survey methods to monitor populations of wide-ranging low-density carnivores. *Ecol. Evol.* **10**, 3276–3292 (2020).
5. E. Dröge, S. Creel, M. S. Becker, J. M'soka, Spatial and temporal avoidance of risk within a large carnivore guild. *Ecol. Evol.* **7**, 189–199 (2017).
6. E. Dröge, S. Creel, M. S. Becker, J. M'soka, Spatial and temporal avoidance of risk within a large carnivore guild. *Ecol. Evol.* **7**, 189–199 (2017).
7. K. T. Everatt, J. F. Moore, G. I. H. Kerley, Africa's apex predator, the lion, is limited by interference and exploitative competition with humans. *Glob. Ecol. Conserv.* **20**, e00758 (2019).
8. K. Gogoi, U. Kumar, K. Banerjee, Y. V. Jhala, Spatially explicit density and its determinants for Asiatic lions in the Gir forests. *PLoS One*. **15**, 1–19 (2020).
9. D. S. Green, L. Johnson-Ulrich, H. E. Couraud, K. E. Holekamp, Anthropogenic disturbance induces opposing population trends in spotted hyenas and African lions. *Biodivers. Conserv.* **27**, 871–889 (2018).
10. M. W. Hayward, G. J. Hayward, Activity patterns of reintroduced lion Panthera leo and spotted hyaena Crocuta crocuta in the Addo Elephant National Park, South Africa. *Afr. J. Ecol.* **45**, 135–141 (2006).
11. A. J. Loveridge, M. Valeix, N. B. Elliot, D. W. Macdonald, The landscape of anthropogenic mortality: how African lions respond to spatial variation in risk. *J. Appl. Ecol.* **54**, 815–825 (2017).
12. T. M. Maddox, thesis (2003).
13. J. R. B. Miller, R. T. Pitman, G. K. H. Mann, A. K. Fuller, G. A. Balme, Lions and leopards coexist without spatial, temporal or demographic effects of interspecific competition. *J. Anim. Ecol.* **87**, 1709–1726 (2018).
14. K. L. Mills, Y. Harissou, I. T. Gnoumou, Y. I. Abdel-Nasseer, B. Doamba, N. C. Harris, Comparable space use by lions between hunting concessions and

national parks in West Africa. *J. Appl. Ecol.* **57**, 975–984 (2020).

15. K. L. Mills, N. C. Harris, Humans disrupt access to prey for large African carnivores. *Elife*. **9**, e60690 (2020).

16. N. L. Mogensen, J. O. Ogutu, T. Dabelsteen, N. L. Mogensen, J. O. Ogutu, T. Dabelsteen, N. L. Mogensen, J. O. Ogutu, T. Dabelsteen, The effects of pastoralism and protection on lion behaviour, demography and space use in the Mara Region of Kenya. *African Zool.* **46**, 78–87 (2011).

17. A. Oriol-Cotterill, D. W. Macdonald, M. Valeix, S. Ekwanga, L. G. Frank, Spatiotemporal patterns of lion space use in a human-dominated landscape. *Anim. Behav.* **101**, 27–39 (2015).

18. S. L. Schooler, S. P. Finnegan, N. L. Fowler, K. F. Kellner, A. L. Lutto, J. Parchizadeh, M. Van Den Bosch, A. Z. Perez, L. M. Masinde, S. B. Mwampeta, H. M. Boone, M. G. Gantchoff, J. E. Hill, T. M. Kautz, N. H. Wehr, R. Fyumagwa, J. L. Belant, Factors influencing lion movements and habitat use in the western Serengeti ecosystem, Tanzania. *Sci. Rep.* **12**, 18890 (2022).

19. P. Schuette, A. P. Wagner, M. E. Wagner, S. Creel, Occupancy patterns and niche partitioning within a diverse carnivore community exposed to anthropogenic pressures. *Biol. Conserv.* **158**, 301–312 (2013).

20. C. E. Searle, J. B. Smit, J. J. Cusack, P. Strampelli, A. Grau, L. Mkuburo, D. W. Macdonald, A. J. Loveridge, A. J. Dickman, Temporal partitioning and spatiotemporal avoidance among large carnivores in a human-impacted African landscape. *PLoS One*. **16**, 1–20 (2021).

21. G. Spong, Space use in lions, Panthera leo, in the Selous Game Reserve: Social and ecological factors. *Behav. Ecol. Sociobiol.* **52**, 303–307 (2002).

22. J. P. Suraci, L. G. Frank, A. Oriol-Cotterill, S. Ekwanga, T. M. Williams, C. C. Wilmers, Behavior-specific habitat selection by African lions may promote their persistence in a human-dominated landscape. *Ecology*. **100**, e02644 (2019).

23. M. Valeix, G. Hemson, A. J. Loveridge, G. Mills, D. W. Macdonald, Behavioural adjustments of a large carnivore to access secondary prey in a human-dominated landscape. *J. Appl. Ecol.* **49**, 73–81 (2012).

24. L. K. Van Der Weyde, C. Mbisana, R. Klein, Multi-species occupancy modelling of a carnivore guild in wildlife management areas in the Kalahari. *Biol. Conserv.* **220**, 21–28 (2018).

Appendix A: Web of Science and ProQuest full search strings used in original literature search (Identification step in Figure S1).

ISI Web of Science Search string:

```
“(    TS = (("Panthera leo" OR "African lion" OR "Lion") AND (human OR
anthropogenic)
      AND
      (Avoid* OR "Space use" OR Spatial OR Respon* OR Behavior OR
      temporal OR diel OR "Land use" OR Management)
      NOT “mountain lion” NOT "primate*" NOT "tamarin")
AND
      WC = (ECOLOGY OR BIODIVERSITY CONSERVATION OR ZOOLOGY OR
      ENVIRONMENTAL SCIENCES OR MULTIDISCIPLINARY SCIENCES OR
      VETERINARY SCIENCES OR BEHAVIORAL SCIENCES OR BIOLOGY OR
      EVOLUTIONARY BIOLOGY OR ENVIRONMENTAL STUDIES)
AND
      DOCUMENT TYPES: (Article)
```

) ” *(end of search string)*

Timespan=1990-2021

ProQuest Dissertations & Theses database search string:

```
“(    (ab("Panthera leo" OR "African lion" OR "Lion"
      NOT ("primat*" OR "tamarin*" OR "mountain lion" OR "pinniped*" OR
      "sea lion" OR "puma"))
      AND (human OR anthropogenic) AND (Avoid* OR "Space use" OR
      Spatial OR Respon* OR Behavior OR temporal OR diel OR "Land use"
      OR Management)
STYPE(dissertations theses)
AND
      subt.exact("ecology" OR "wildlife conservation" OR "zoology" OR
      "wildlife management" OR "conservation" OR "environmental
      science" OR "recreation" OR "agricultural economics" OR
      "behavioral sciences" OR "forestry" OR "environmental studies" OR
      "management" OR "veterinary services")
AND
      pd(>19901231)
```

) ” *(end of search string*
