# Supplementary material for: Tradeoffs between resources and risks shape the responses of a large carnivore to human disturbance
Source: Commun Biol. 2023 Oct 17;6:986. doi: 10.1038/s42003-023-05321-z (PMC10582050; doi:10.1038/s42003-023-05321-z)
Supplement: Supplementary file 3 — Description of Additional Supplementary Files [file 42003_2023_5321_MOESM3_ESM.pdf]

Description of Additional Supplementary Files

File name: Supplementary Data

Description: The source data for all statistical analyses in the paper.
