# Supplementary material for: Tradeoffs between resources and risks shape the responses of a large carnivore to human disturbance
Source: Commun Biol. 2023 Oct 17;6:986. doi: 10.1038/s42003-023-05321-z (PMC10582050; doi:10.1038/s42003-023-05321-z)
Supplement: Supplementary file 5 — Reporting Summary [file 42003_2023_5321_MOESM5_ESM.pdf]

nature portfolio

Corresponding author(s): Kirby L. Mills

Last updated by author(s): Aug 26, 2023

Reporting Summary

Nature Portfolio wishes to improve the reproducibility of the work that we publish. This form provides structure for consistency and transparency in reporting. For further information on Nature Portfolio policies, see our Editorial Policies and the Editorial Policy Checklist.

Statistics

For all statistical analyses, confirm that the following items are present in the figure legend, table legend, main text, or Methods section.

n/a	Confirmed	
☐	☒	The exact sample size (*n*) for each experimental group/condition, given as a discrete number and unit of measurement
☒	☐	A statement on whether measurements were taken from distinct samples or whether the same sample was measured repeatedly
☐	☒	The statistical test(s) used AND whether they are one- or two-sided<br>*Only common tests should be described solely by name; describe more complex techniques in the Methods section.*
☐	☒	A description of all covariates tested
☐	☒	A description of any assumptions or corrections, such as tests of normality and adjustment for multiple comparisons
☐	☒	A full description of the statistical parameters including central tendency (e.g. means) or other basic estimates (e.g. regression coefficient) AND variation (e.g. standard deviation) or associated estimates of uncertainty (e.g. confidence intervals)
☐	☒	For null hypothesis testing, the test statistic (e.g. *F*, *t*, *r*) with confidence intervals, effect sizes, degrees of freedom and *P* value noted<br>*Give P values as exact values whenever suitable.*
☒	☐	For Bayesian analysis, information on the choice of priors and Markov chain Monte Carlo settings
☒	☐	For hierarchical and complex designs, identification of the appropriate level for tests and full reporting of outcomes
☐	☒	Estimates of effect sizes (e.g. Cohen's *d*, Pearson's *r*), indicating how they were calculated

Our web collection on statistics for biologists contains articles on many of the points above.

Software and code

Policy information about availability of computer code

Data collection	No software was used to collect the data.
Data analysis	All data analysis was completed using R language in RStudio open source software. No novel code was used.

For manuscripts utilizing custom algorithms or software that are central to the research but not yet described in published literature, software must be made available to editors and reviewers. We strongly encourage code deposition in a community repository (e.g. GitHub). See the Nature Portfolio guidelines for submitting code & software for further information.

Data

Policy information about availability of data

All manuscripts must include a data availability statement. This statement should provide the following information, where applicable:

- Accession codes, unique identifiers, or web links for publicly available datasets
- A description of any restrictions on data availability
- For clinical datasets or third party data, please ensure that the statement adheres to our policy

Data necessary for the analyses in this study will be made available on Dryad data repository upon publication, including the study and site characteristics, extracted measures of lion spatiotemporal activity and their calculated effect sizes, and spatial covariates used in the meta-regression models. The code used in our analysis is not novel code.

Human research participants

Policy information about studies involving human research participants and Sex and Gender in Research.

Reporting on sex and gender	N/A
Population characteristics	N/A
Recruitment	N/A
Ethics oversight	N/A

Note that full information on the approval of the study protocol must also be provided in the manuscript.

Field-specific reporting

Please select the one below that is the best fit for your research. If you are not sure, read the appropriate sections before making your selection.

☐ Life sciences ☐ Behavioural & social sciences ☒ Ecological, evolutionary & environmental sciences

For a reference copy of the document with all sections, see nature.com/documents/nr-reporting-summary-flat.pdf

Ecological, evolutionary & environmental sciences study design

All studies must disclose on these points even when the disclosure is negative.

Study description	To investigate how lions navigate shared human-natural landscapes, we conducted a meta-analysis that examines lion spatiotemporal responses to human disturbance over gradients of disturbance intensity and resource availability.
Research sample	We compiled data on lion observations of spatiotempoal activity or results from studies using lion activity data from 31 independent study sites across the range of lions. The study sample is at the continental scale, with data ranging from 1994 to 2019. Some data was pulled directly form published scientific literature or published datasets, while others were provided as raw data from study authors.
Sampling strategy	We extracted as many data points as possible for each study, but used a subset of data representing the extremes of human disturbance for studies where N > 60 for low and high disturbance categories. N = 60 as a cutoff was selected using a power analysis to determine the minimum number of samples per group that would provide adequate power to detect moderate effects.
Data collection	Data collection began with a systematic literature review, including snowball searching, with inclusion criteria that are described in the text. Lion activity data was extracted from published literature or solicited from study authors for all relevant publications.
Timing and spatial scale	Data collection began on 2021/05/07 and ended on approximately 2022/01/30. The spatial extent of the study spans all of Africa and also includes Gir Protected Area in India.
Data exclusions	Relevant studies were excluded only when we were unable to obtain activity data from the publications and authors. No activity data that we extracted or received from study authors were excluded from our analyses.
Reproducibility	To ensure reproducibility we have included comprehensive descriptions of the included studies and how we extracted and processed data from each study, along with the resulting effect size calculations for each study. With the information we provided a reader is able to locate studies, extract data, and compare results with our study results. Our data analyses are described in detail and use widely available methods and softwares that enable reproducing our results.
Randomization	Randomization does not apply to our meta-analysis framework because study-specific design was not in our control.
Blinding	Blinding does not apply to our meta-analysis framework because we used already published and vetted data and results from individual studies.

Did the study involve field work? ☐ Yes ☒ No

Reporting for specific materials, systems and methods

We require information from authors about some types of materials, experimental systems and methods used in many studies. Here, indicate whether each material, system or method listed is relevant to your study. If you are not sure if a list item applies to your research, read the appropriate section before selecting a response.

Materials & experimental systems

n/a	Involved in the study
☒	☐ Antibodies
☒	☐ Eukaryotic cell lines
☒	☐ Palaeontology and archaeology
☒	☐ Animals and other organisms
☒	☐ Clinical data
☒	☐ Dual use research of concern

Methods

n/a	Involved in the study
☒	☐ ChIP-seq
☒	☐ Flow cytometry
☒	☐ MRI-based neuroimaging
