# Supplementary material for: Tradeoffs between resources and risks shape the responses of a large carnivore to human disturbance
Source: Commun Biol. 2023 Oct 17;6:986. doi: 10.1038/s42003-023-05321-z (PMC10582050; doi:10.1038/s42003-023-05321-z)
Supplement: Supplementary file 6 — Supplementary Data 1 - README [file 42003_2023_5321_MOESM6_ESM.docx]

File name: LionHuman_Meta-Analysis.xlsx

Creator: Kirby Mills (kimills@umich.edu)

Other personnel: Indira Sankaran (insankar@umich.edu); Nate Sanders (njsand@umich.edu): Neil Carter (nhcarter@umich.edu)

**Workbook Tab Descriptions:**

*Study Summaries*: Characteristics of each study identified that might have usable data, including the study site and methods attributes, a description of what data is available from publication, classifications of usability (low, med, high) for the available data, progress trackers (updated as we go), and notes. This sheet includes any publications from the “*New papers”* and “*WoS Search Results”* tabs that were evaluated as usable at the full-text stage.

*Data Entry_spatial*: Data extraction spreadsheet to be used in further analysis. Data here is only for lions’ spatial responses to human disturbances. Similar to *Data Entry_temporal* tab with a few different columns*.*

*Data Entry_temporal*: Data extraction spreadsheet to be used in further analysis. Data here is only for lions’ temporal responses to human disturbances. Similar to *Data Entry_spatial* tab with a few different columns*.*

*Author contact list:* List of authors identified to contact to request unpublished data sets (noted in *Study Summaries* tab). Progress trackers (updated as we go, in yellow), author names, emails, and study info used to populate emails.

*New papers:* List of publications identified from snowball searching reference lists, evaluation of abstracts and full text. If full-text evaluation was ‘yes’, the study was added to the “*Study summaries”* tab

*WoS Search Results – title filt:* List of publications returned by the Web of Science search, excluding those that were deemed unfit by the title alone. See “*Original Paper Set from WoS*” for full set of results. If full-text evaluation was ‘yes’, the study was added to the “*Study summaries”* tab

*Original Paper Set from WoS:*  Full list of publications returned by the Web of Science search. Red text indicates publications that were excluded because of their title

*Interesting papers:* Publications identified during literature seaerch by KM that seemed interesting

**Worksheet column descriptions:**

*unused columns that will eventually be deleted but deleting things make me anxious

***Tab: Data Entry_spatial***

Data input by – initials of person who input the row of data (first, middle, last)

ID – the ID of the study and sub-ID of the effect calculation (e.g., 03-02 is study ID 03 and the 2^nd^ effect size to be calculated in that study)

Study – Author name and year

Study Area (SA) – Name of the study area

Sub-SA – Name of the sub-study area, if relevant (i.e., if lion responses are calculated for more than one site in a study)

SA size – size of the study area in square kilometers

SA type – management type for the study area

Coord type – are the coordinates provided as a centroid or range of coordinates?

Latitude – latitude coordinates of SA (separated by comma if a range)

Longitude – longitude coordinates of SA (separated by comma if a range)

Season – The season in which the study took place (wet, dry, year-round, or range of months)

Months – duration of the study in number of months

Country – country in which the study took place

Continent – continent in which the study took place

Subset of – If the calculated effect size use da subset of data included in another ES calculation, the ID of that effect size

Data type – type of data collected in the study (camera trap, telemetry, observation, tracks, etc.)

Sample unit – The scale at which individual data points are recorded (camera, grid_cell, transect, etc.)

Sampling effort – Sampling effort used to collect the data

N lions – number of lions observed for this effect size (not relevant to camera trap or transect data)

Human disturbance metric – variable/metric used to measure human disturbance for this ES calculation

Human disturbance comparison – How levels of human disturbance were compared; between discrete locations/sites [location (discrete)], within a mosaic of heterogeneous disturbance levels [locations (mosaic)], between times of different disturbance levels (temporal).

Human disturbance scale – How human disturbance was measured, as a continuous or discrete variable

Lion response type – spatial vs. temporal (should all be spatial for this sheet)

Lion use metric – variable/metric used to measure lion activity

Sample diel period – over what times of day were the data collected (nocturnal, diurnal, crepuscular, 24h)

Lion sex – the sex of lions being observed (male, female, both)

Data_source – the source within the publication of the data being extracted to calculate the ES (figure number, supporting info number, raw_data)

x_high – the average/mean of lion activity/use in areas of high human disturbance

sd_high – the standard deviation of lion activity/use in areas of high human disturbance

n_high – the sample size for calculating the mean/sd of lion activity/use in areas of high human disturbance

x_low – the average/mean of lion activity/use in areas of low human disturbance

sd_low – the standard deviation of lion activity/use in areas of low human disturbance

n_low – the sample size for calculating the mean/sd of lion activity/use in areas of low human disturbance

Data extraction description – Written description of how the data were extracted from the publication or raw data, including how we designated low and high human disturbance observations

Full citation – study citation in APA (copy from Google Scholar for consistency)

***Tab: Data Entry_temporal*** *- SAME AS ABOVE EXCEPT FOR THE LISTED COLUMNS*

Lion response type – spatial vs. temporal (should all be temporal for this sheet)

prop-noct_high – the calculated proportion of nocturnal lion activity in areas of high human disturbance

*sd_high – the standard deviation of lion nocturnality in areas of high human disturbance

n_high – the sample size for calculating the proportion/sd of lion nocturnality in areas of high human disturbance

prop-noct _low – the calculated proportion of nocturnal lion activity in areas of low human disturbance

*sd_low – the standard deviation of lion nocturnality in areas of low human disturbance

n_low – the sample size for calculating the proportion/sd of lion nocturnality in areas of low human disturbance
